# Separate mechanisms regulating accumbal taurine levels during baseline conditions and following ethanol exposure in the rat

**DOI:** 10.1038/s41598-024-74449-7

**Published:** 2024-10-15

**Authors:** Karin Ademar, Lisa Ulenius, Anna Loftén, Bo Söderpalm, Louise Adermark, Mia Ericson

**Affiliations:** 1 Addiction Biology Unit, Department of Psychiatry and Neurochemistry, Institute of Neuroscience and Physiology, Sahlgrenska Academy, University of Gothenburg, Box 410, 405 30 Gothenburg, Sweden; 2 Department of Pharmacology, Institute of Neuroscience and Physiology, Sahlgrenska Academy, University of Gothenburg, Gothenburg, Sweden; 3 Beroendekliniken, Sahlgrenska University Hospital, Gothenburg, Sweden

**Keywords:** Ion channels in the nervous system, Neural circuits, Neuronal physiology, Reward, Synaptic transmission

## Abstract

Ethanol-induced dopamine release in the nucleus accumbens (nAc) is associated with reward and reinforcement, and for ethanol to elevate nAc dopamine levels, a simultaneous increase in endogenous taurine is required within the same brain region. By employing in vivo microdialysis in male Wistar rats combined with pharmacological, chemogenetic and metabolic approaches, our aim with this study was to identify mechanisms underlying ethanol-induced taurine release. Our results demonstrate that the taurine elevation, elicited by either systemic or local ethanol administration, occurs both in presence and absence of action potential firing or NMDA receptor blockade. Inhibition of volume regulated anion channels did not alter the ethanol-induced taurine levels, while inhibition of the taurine transporter occluded the ethanol-induced taurine increase, putatively due to a ceiling effect. Selective manipulation of nAc astrocytes using G_q_-coupled designer receptors exclusively activated by designer drugs (DREADDs) did not affect ethanol-induced taurine release. However, activation of G_i_-coupled DREADDs, or metabolic inhibition using fluorocitrate, rather enhanced than depressed taurine elevation. Finally, ethanol-induced taurine increase was fully blocked in rats pre-treated with the L-type Ca^2+^-channel blocker nicardipine, suggesting that the release is Ca^2+^ dependent. In conclusion, while astrocytes appear to be important regulators of basal taurine levels in the nAc, they do not appear to be the main cells underlying ethanol-induced taurine release.

**Supplementary Information:**

The online version contains supplementary material available at 10.1038/s41598-024-74449-7.

## Introduction

The endogenous amino acid taurine has a number of physiological functions throughout the body^1^. In the brain, taurine can be found in both neurons and glial cells, and the concentration appears to depend on the brain region studied^2–5^. One of the most studied properties of taurine is its involvement in osmoregulation^6^. When glia cells and neurons swell in response to changes in osmotic pressure, or due to other factors, they release inorganic ions and organic solutes such as taurine, glycine and glutamate as part of re-equilibration of the osmotic imbalance, a cell-intrinsic process known as regulatory volume decrease (RVD)^7–11^. A major contributor to the osmotically driven taurine efflux is the volume regulated anion channels (VRACs), which can be found on most neuronal cells in comparable levels^1,11–14^. It is not until recently that the molecular identity of VRACs has been recognized as heterohexameres belonging to the leucine-rich repeat-containing 8 (LRRC8) protein family^15,16^, and in rat astrocytes, the LLRC8A-containing VRACs have been linked to swelling activated release of taurine^17^. Volume regulation following osmotic swelling appears to be controlled by Ca^2+^ fluxes^18,19^. However, the dependency of Ca^2+^ is found to be highly variable depending on the cell type studied^19^ and in astrocytes, RVD is reported both as Ca^2+^ dependent and Ca^2+^ independent^17,18^.

The mesolimbic dopamine system is a major site of action for the stimulatory effects of ethanol in the brain^20,21^. Ethanol exposure results in elevated dopamine levels in the nucleus accumbens (nAc), which is the brain area suggested to contribute to the rewarding and reinforcing properties of ethanol and other addictive drugs^20–22^. Besides increased dopamine levels in the nAc, an increase in extracellular taurine has also been shown in the same brain region after ethanol administration^23–26^. This increase in taurine levels has further been demonstrated to be necessary for ethanol’s ability to elevate dopamine levels^25^. The release of taurine in response to ethanol appears to be a robust phenomenon, which is not altered following taurine depletion or supplementation^25,27^. Furthermore, as taurine analogs (e.g. acamprosate (Campral^®^), used for relapse prevention in alcohol use disorder) also influence dopaminergic output in the nAc^28^ and reduce ethanol intake^29–31^, it is of importance to understand the mechanisms by which ethanol induces taurine efflux in the nAc. Since it has been shown that ethanol alters the extracellular environment and induces swelling of astrocytes^32–34^, and that taurine is one of the primary osmolytes in astrocytes^35,36^, it is conceivable that astrocytes may release taurine for osmoregulatory purposes as a part of the RVD following ethanol exposure. We thus hypothesize that taurine is released from astrocytes following ethanol exposure in order to re-equilibrate the osmotic imbalance induced by ethanol and that VRACs could be responsible for the ethanol-induced efflux of taurine, as these channels are important in osmotically-dependent release of taurine in cell volume regulation^13,37^. Although LRRC8 proteins also are expressed in neurons, we hypothesize that the ethanol-mediated taurine elevation derives from astrocytes since these cells are more reactive to changes in the extracellular milieu. Thus, the overall aim of this study was to further outline the mechanisms underlying ethanol-induced taurine elevation in the nAc.

## Methods and materials

### Research outline

In the present study, we more specifically aimed to determine whether;^1^ the ethanol-induced elevation of extracellular taurine in the nAc requires action potential-mediated neurotransmission;^2^ the ethanol induced taurine release is mediated via VRACs as a part of the RVD following ethanol exposure;^3^ chemogenetic or metabolic manipulations of astrocytes can influence the ethanol-induced increase in extracellular levels of taurine, and^4^ the ethanol-induced increase of accumbal taurine derives from other cell types and/or is dependent on Ca^2+^. Studies were based on in vivo microdialysis conducted in drug naïve and freely moving male Wistar rats and was complemented with immunofluorescence to verify viral transfection. A detailed description of methods and materials are outlined in supplementary information.

### Animals

Male Wistar rats (*n* = 347) were group housed at regular 12-hour light/dark cycle, from arrival until surgery with food and water *ad libitum*. The experiments were approved by the Ethics Committee for Animal Experiments in Gothenburg, Sweden (2401/19; 3095/20), followed the Swedish National Committee for Animal Research guidelines and is reported in accordance with ARRIVE guidelines.

### Viral microinjection of DREADDs targeting astrocytes

Rats (*n* = 101, 190–210 g) were unilaterally injected with 0.8 µl of the viral vector (pssAAV-2-hGFAP-HA_hM_3_D(Gq)-IRES-mCitrine-WPRE-hGHp(A), pssAAV-5/2-hGFAP-hM_4_D(Gi)_mCherry-WPRE-hGHp(A), or pssAAV-2-hGFAP-EGFP-WPRE-hGHp(A)) into the nAc core/shell borderline region (AP: +1.5 mm, ML: -1.4 mm relative to bregma and DV: -7.8 mm relative to the scull^38^) three weeks before any further procedure.

### In vivo microdialysis

Two days prior to the in vivo microdialysis experiment, rats (280–360 g) were anesthetized by isoflurane and mounted onto a stereotaxic instrument. A dialysis probe was lowered into the nAc (AP: +1.85 mm, ML: -1.4 mm relative to bregma and DV: -7.8 mm relative to dura mater^38^) and fixed to the skull using anchoring screws and dental cement. Animals were single-housed during the 48-hour surgical recovery. On the experimental day, the microdialysis probe was connected to a microperfusion pump and the probe was perfused with Ringer’s solution, for two hours, prior to baseline sampling. Dialysate samples (40 µl) were collected every 20 min. Drug administration was initiated when four stable baseline samples (± 10%) were obtained. After the experiment, brains were removed, fixed, and stored (4 °C) for 4–7 days until probe placement verification (Fig. S1).

### Biochemical assays

High performance liquid chromatography with fluorescence detection was used for separation and detection of taurine. External standards were used for identification and quantification of the taurine concentration. The chromatogram was analyzed using Thermo Scientific Chromeleon Chromatography Data System software (CHROMELEON7).

### Immunofluorescence

Brains were post fixed, cryoprotected and snap-frozen on dry ice. Coronal slices (40 μm) were sectioned using a cryostat and placed in cryoprotective medium (-20 °C). Brain sections were washed, blocked and incubated with primary antibody overnight (4 °C). Sections were washed and incubated with secondary antibody for one hour (room temperature; RT). Sections were washed, mounted onto microscope slides, coverslipped and dried overnight (RT). Images were obtained with a Zeiss LSM 700 inverted confocal microscope.

### Statistics

Data were analyzed using GraphPad Prism, version 10 for Windows. Statistical significance was evaluated using two-way analysis of variance (ANOVA) over the relevant time period followed by Tukey’s post hoc analysis, one-way ANOVA over the relevant time period followed by Tukey’s post hoc analysis or unpaired *t*-test. The relevant time period was based on when the drug used for pre-treatment yielded stable taurine levels, which then was decisive for when ethanol was to be administered. All data are presented as mean ± standard error of the mean (SEM) and a probability value (*p*) less than 0.05 was considered statistically significant.

## Results

### Ethanol increases nAc taurine levels following different routes of administration

**Fig. 1:**
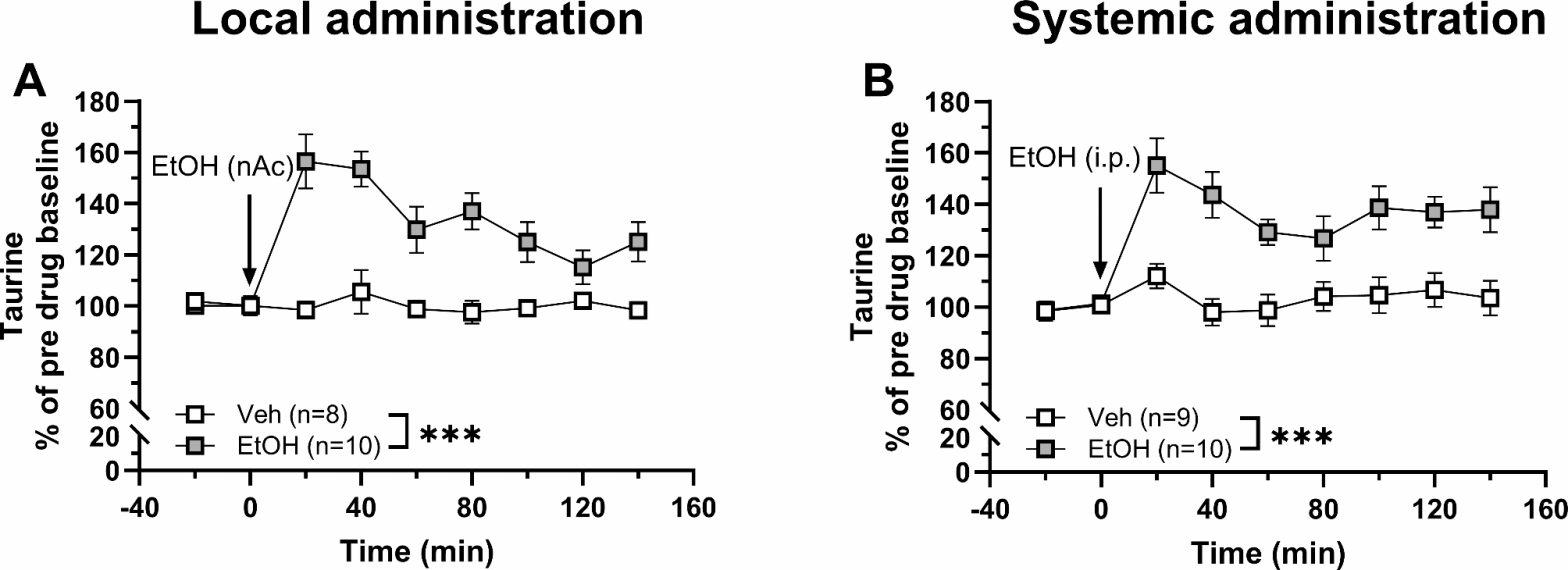
Local and systemic administration of ethanol increase taurine levels in the nAc. Time-course graphs of extracellular levels of taurine in the nAc after **A** local administration of ethanol (300 mM, reversed microdialysis) or vehicle (Ringer’s solution) and **B** systemic administration of ethanol (2.5 g/kg, i.p.) or vehicle (0.9% NaCl, i.p.). The extracellular taurine levels were significantly elevated following both routes of ethanol administration compared to vehicle-treated controls. The cut in the y-axis is present to improve the visual effect of the result. All data are presented as mean ± SEM. ***p < 0.001. EtOH = ethanol, nAc = nucleus accumbens, Veh = vehicle.

Compared to vehicle-treated control, administration of ethanol (EtOH) significantly increased the extracellular levels of taurine after both local perfusion (300 mM, nAc; two-way ANOVA_t=0–140 min_: group effect *F*_(1, 16)_ = 21.64, *p* < 0.001, time effect: *F*_(4.14, 66.22)_ = 8.09, *p* < 0.001, interaction between the treatment groups over time: *F*_(8, 128)_ = 8.09, *p* < 0.001; Fig. 1A) and systemic administration (2.5 g/kg, 15 ml/kg, i.p.; two-way ANOVA_t=0–140 min_: group effect: *F*_(1, 17)_ = 16.50, *p* < 0.001, time effect: *F*_(3.78, 64.33)_ = 9.35, *p* < 0.001, interaction between the groups over time: *F*_(8, 136)_ = 5.33, *p* < 0.001; Fig. 1B).

### Tetrodotoxin administration does not prevent ethanol-induced elevation of taurine levels

**Fig. 2:**
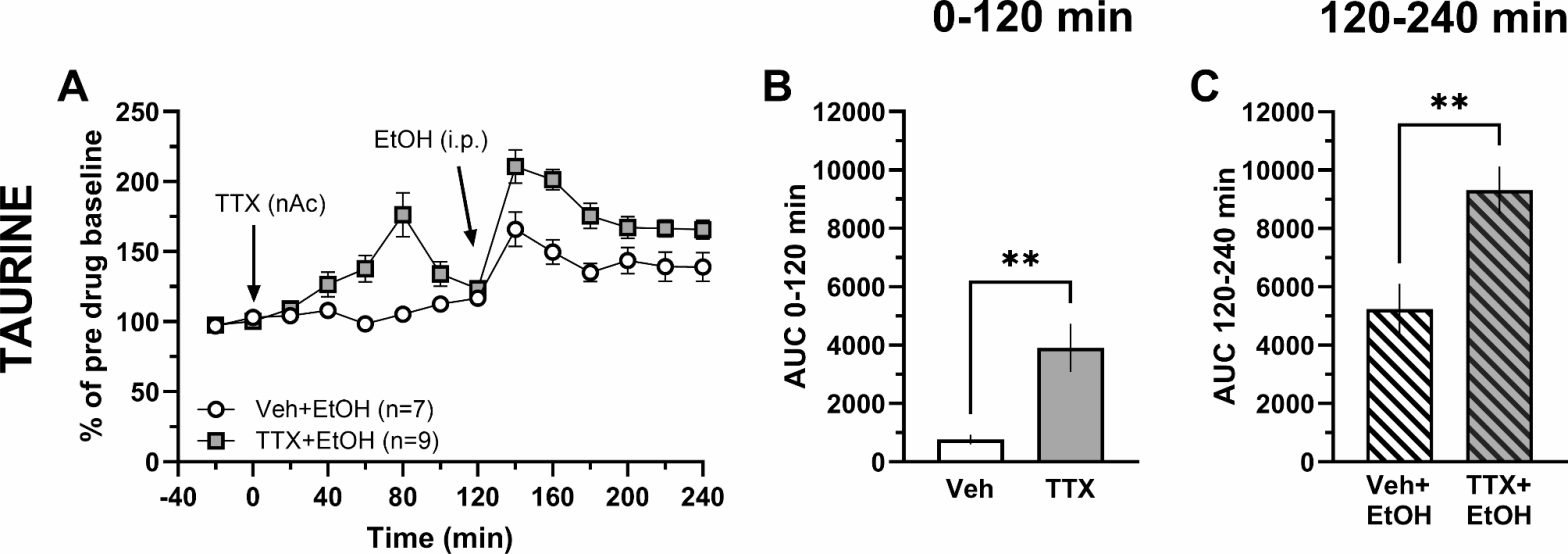
Tetrodotoxin does not prevent ethanol from increasing taurine in the nAc. Time-course graphs of extracellular levels of **A** taurine in the nAc after TTX (1 µM, reversed microdialysis) and ethanol (2.5 g/kg i.p.) administration. Area under the curve of **B** taurine levels during local perfusion with vehicle (Ringer’s solution) or TTX during 0–120 min demonstrates that TTX increases extracellular taurine levels compared to Ringer-treated controls. Area under the curve of **C** taurine levels during local perfusion with vehicle or TTX and the addition of ethanol (i.p.) during 120–240 min demonstrates that ethanol further elevates extracellular taurine levels in TTX-perfused animals. All data are presented as mean ± SEM. **p < 0.01. AUC = area under the curve, EtOH = ethanol, nAc = nucleus accumbens, TTX = tetrodotoxin, Veh = vehicle.

To examine whether the ethanol-induced elevation of taurine requires action potential firing, the Na^+^-channel blocker tetrodotoxin (TTX; 1 µM) was perfused in the nAc followed by a systemic injection of ethanol (2.5 g/kg, i.p.). Administration of TTX alone elevated taurine levels compared to vehicle treatment (*t*-test of AUC_t=0–120 min_: *t*_14_ = 3.30, *p* = 0.005; Fig. 2A-B). Furthermore, while ethanol increased extracellular levels of taurine both in the presence and absence of TTX, the relative increase was significantly greater following TTX (*t*-test of AUC_t=120–240 min_: *t*_14_ = 3.41, *p* = 0.004; Fig. 2A, C), suggesting that action potential firing both acts to reduce basal taurine levels under normal baseline conditions, as well as suppresses ethanol-induced taurine elevation.

### NMDA receptor blockade using memantine does not inhibit ethanol-induced taurine release

**Fig. 3:**
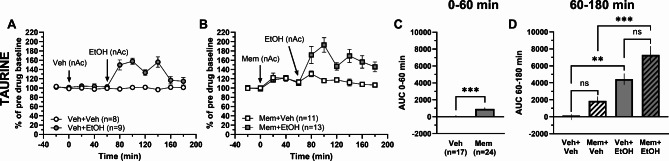
Local administration of memantine increases taurine levels. Time-course graphs of extracellular levels of **A** taurine in the nAc after administration of ethanol (300 mM) or vehicle (Ringer’s solution) within the nAc using reversed microdialysis. Extracellular levels of **B** taurine in the nAc following local administration of the NMDA receptor antagonist memantine (100 µM) alone or together with ethanol. Area under the curve of **C** taurine levels during local perfusion with vehicle or memantine during 0–60 min demonstrates memantine to elevate extracellular taurine levels compared to Ringer-treated controls. Area under the curve of **D** taurine levels during local perfusion with vehicle, ethanol or memantine alone or together with ethanol in the nAc during time point 60–180 min shows that ethanol increases extracellular taurine levels compared to Ringer-treated controls and that memantine combined with ethanol increase extracellular taurine levels compared to memantine alone. All data are presented as mean ± SEM. **p < 0.01, ***p < 0.001. AUC = area under the curve, EtOH = ethanol, Mem = memantine, nAc = nucleus accumbens, Veh = vehicle.

NMDA receptor agonists have previously been shown to evoke taurine release^39^, and inhibition of the NMDA receptor using MK801 prevents ethanol-induced taurine efflux in the hippocampus^40^. Since the NMDA receptor antagonist memantine has been demonstrated to attenuate relapse-like drinking behavior in the rat in the alcohol deprivation model^41^, memantine was perfused alone and in combination with ethanol within the nAc. Local administration of memantine (Mem; 100 µM) elevated accumbal taurine levels compared to vehicle-treated animals (*t*-test of AUC_t=0–60 min_: *t*_39_ = 4.01, *p* < 0.001; Fig. 3A-C). Ethanol (300 mM) perfusion in the nAc increased accumbal taurine levels in naïve- (one-way ANOVA of AUC_t=60–180 min_: *F*_(3, 37)_ = 16.19, *p* < 0.001, *post hoc* analysis: Veh + EtOH vs. Veh + Veh *p* = 0.005; Fig. 3A, D) and memantine-pretreated rats compared to vehicle and memantine treatment alone, respectively (*post hoc* analysis: Mem + EtOH vs. Mem + Veh *p* < 0.001, Veh + Veh vs. Mem + Veh *p* = 0.395, Mem + EtOH vs. Veh + EtOH *p* = 0.066; Fig. 3B, D).

### Blockade of the taurine transporter increases nAc taurine levels with no further elevation mediated by ethanol

**Fig. 4:**
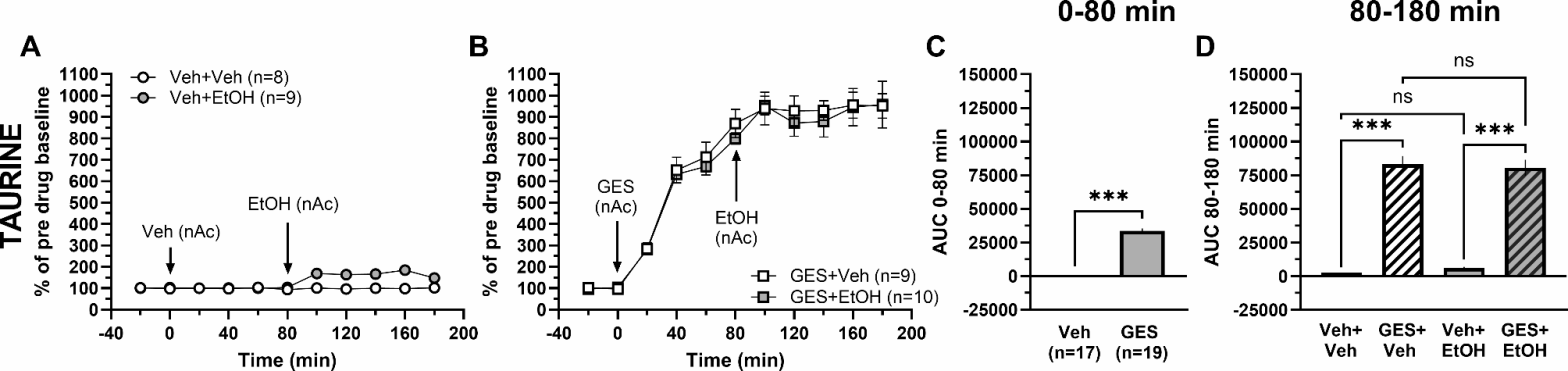
Taurine transport inhibition elevates accumbal taurine levels with no further effect of ethanol. Time-course graphs of extracellular levels of taurine in the nAc following administration of **A** ethanol (300 mM) or vehicle (Ringer’s solution) and **B** the taurine transporter inhibitor GES (5 mM) alone or together with ethanol using reversed microdialysis within the nAc. Area under the curve of **C** taurine levels during local perfusion with vehicle or GES during 0–80 min demonstrates GES to elevate extracellular taurine levels compared to Ringer-treated controls. Area under the curve of **D** taurine levels during local perfusion with vehicle, ethanol or GES alone or together with ethanol in the nAc during time point 80–180 min shows ethanol to increase extracellular taurine levels compared to Ringer-treated controls and GES combined with ethanol not to increase extracellular taurine levels compared to GES alone. All data are presented as mean ± SEM. ***p < 0.001. AUC = area under the curve, EtOH = ethanol, GES = guanidinoethyl sulfonate, nAc = nucleus accumbens, Veh = vehicle.

Taurine is actively transported from the extracellular space into cells, both into neurons and astrocytes, via the taurine transporter^1^. To investigate if blockade of the influx of taurine into cells would affect ethanol-mediated taurine release, we used the taurine transporter blocker guanidinoethyl sulfonate (GES)^6^. Accumbal perfusion of GES (5 mM) substantially increased the taurine levels compared to vehicle treatment (*t*-test of AUC_t=0–80 min_: *t*_34_ = 17.27, *p* < 0.001; Fig. 4A-C). Addition of ethanol (300 mM) locally in the nAc to GES-pretreated rats produced no further increase in accumbal taurine levels compared to GES treatment alone (one-way ANOVA of AUC_t=80–180 min_: *F*_(3, 32)_ = 101.1, *p* < 0.001, *post hoc* analysis: GES + EtOH vs. GES + Veh *p* = 0.972; Fig. 4B, D). The large taurine output found in the nAc after GES-pretreatment resulted in a significant difference between pretreated and naïve rats perfused with ethanol (*post hoc* analysis: GES + EtOH vs. Veh + EtOH *p* < 0.001, Veh + Veh vs. GES + Veh *p* < 0.001; Fig. 4A-B, D), and was most likely the reason for why no significant difference was found between the two groups of naïve rats (*post hoc* analysis: Veh + EtOH vs. Veh + Veh *p* = 0.772; Fig. 4A, D).

### Antagonism of VRACs partly attenuate ethanol-induced elevation of nAc taurine

**Fig. 5:**
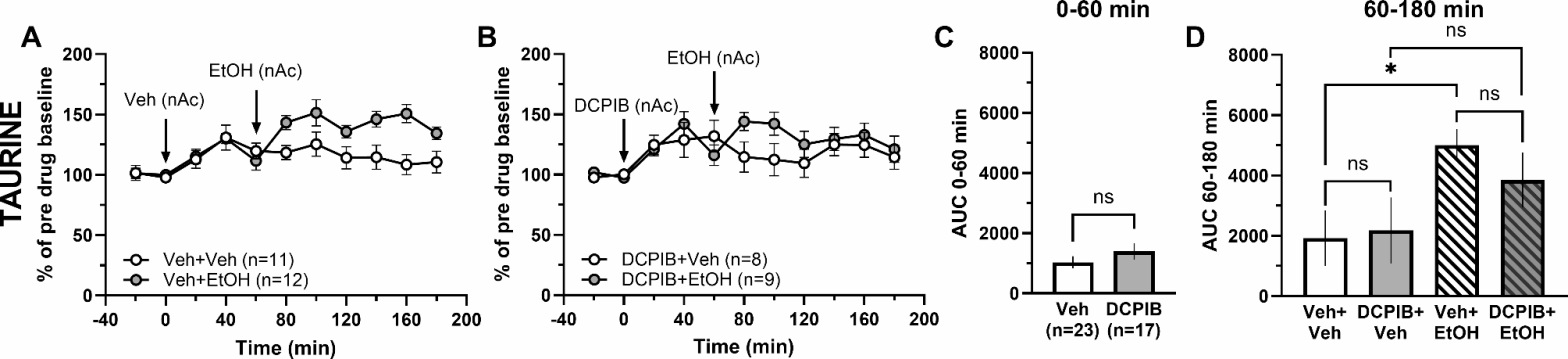
Local perfusion with DCPIB does not decrease ethanol-induced taurine release. Time-course graphs of extracellular levels of taurine in the nAc after administration of **A** ethanol (300 mM) or vehicle (Ringer’s solution + 0.4% DMSO) and **B** the VRAC inhibitor DCPIB (100 µM) alone or together with ethanol using reversed microdialysis within the nAc. **C** Area under the curve of taurine levels during local perfusion with vehicle or DCPIB during 0–60 min demonstrates that DCPIB does not to elevate extracellular taurine levels compared to Ringer + DMSO-treated controls. **D** Area under the curve of taurine levels during local perfusion with vehicle, ethanol or DCPIB alone or together with ethanol in the nAc during time point 60–180 min shows ethanol to increase extracellular taurine levels compared to Ringer + DMSO-treated controls, but DCPIB-treatment does not significantly decrease taurine levels after ethanol application compared to ethanol treatment alone. All data are presented as mean ± SEM. *p < 0.05. AUC = area under the curve, EtOH = ethanol, nAc = nucleus accumbens, Veh = vehicle, VRAC = volume regulated anion channel.

Taurine may also be released through VRACs in a way to induce RVD, thus the VRAC antagonist DCPIB was used to evaluate the role of these channels in ethanol-induced elevation of taurine. Accumbal perfusion of DCPIB (100 µM) had no significant impact on extracellular levels of taurine compared to vehicle treatment (vehicle containing 0.4% DMSO; *t*-test of AUC_t=0–60 min_: *t*_38_ = 1.09, *p* = 0.283; Fig. 5A-C). When ethanol (300 mM) was co-perfused, the accumbal taurine levels of DCPIB-pretreated animals were not significantly altered compared to the vehicle-treated controls (one-way ANOVA of AUC_t=60–180 min_: *F*_(3, 36)_ = 3.20, *p* = 0.035; *post hoc* analysis: DCPIB + EtOH vs. DCPIB + Veh *p* = 0.576, DCPIB + EtOH vs. Veh + EtOH *p* = 0.761, Veh + EtOH vs. Veh + Veh *p* = 0.042, Veh + Veh vs. DCPIB + Veh *p* = 9.997; Fig. 5A-B, D).

### Chemogenetic inhibition of astrocytes using Gi-DREADDs potentiates the ethanol-induced elevation of taurine

**Fig. 6:**
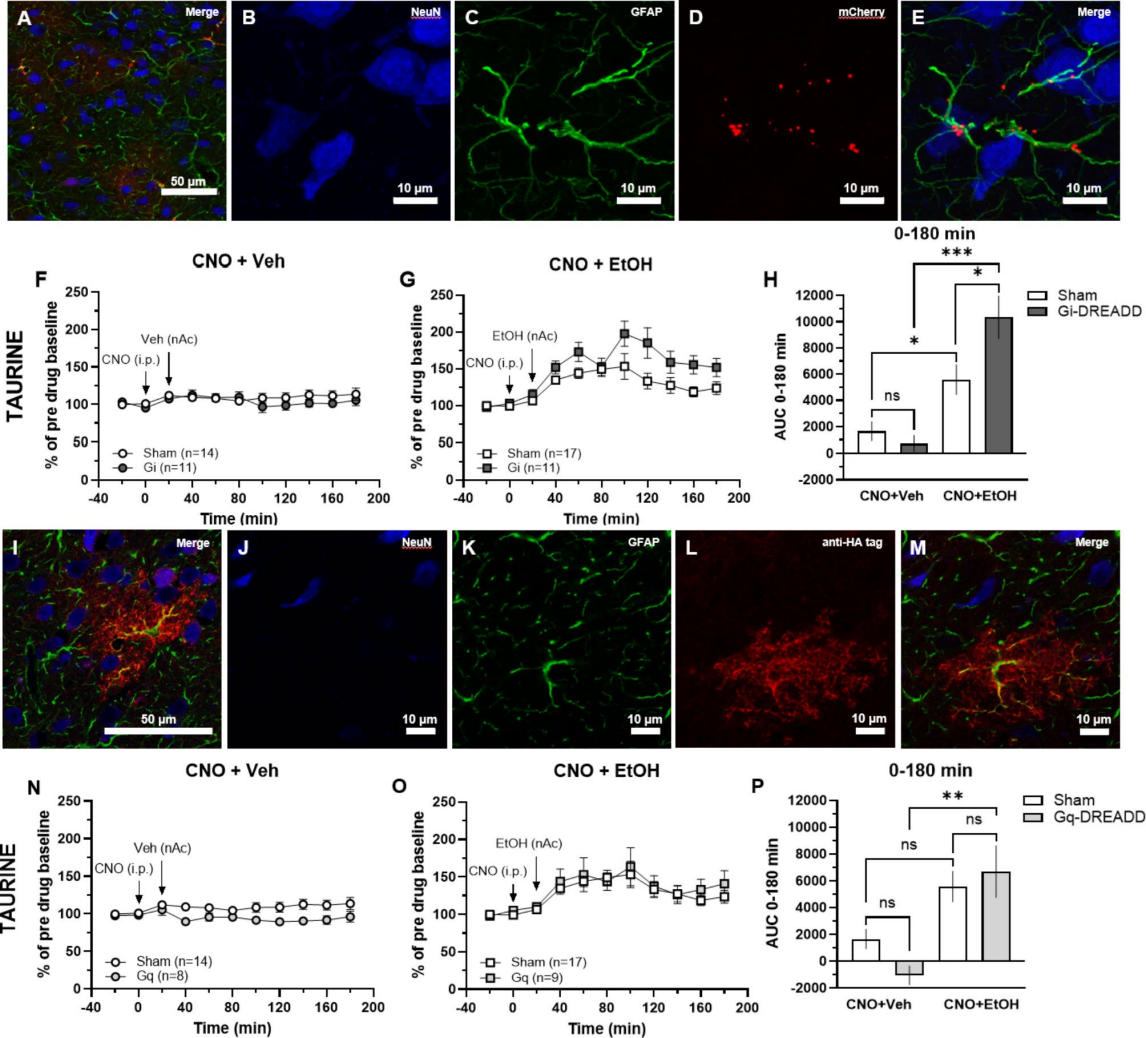
Application of CNO to astrocyte-specific Gi-DREADDs potentiates ethanol-induced taurine elevation, a phenomenon not found using Gq-DREADDs. **A-E** Immunofluorescence images of pssAAV-5/2-hGFAP-hM_4_D(Gi)_mCherry-WPRE-hGHp(A) expression in the nAc. **A** Immunofluorescence staining showing neurons visualized by NeuN in blue, astrocytes expressing GFAP in green, and the DREADD conjugated with mCherry in red (20x, scale bar 50 μm). **B-E  ***From left to right*: NeuN, GFAP, mCherry, and a merge of all three in higher magnification showing mCherry to be associated with cells expressing GFAP and co-localization of GFAP and mCherry (z-stack 63x, scale bar 10 μm). **F** Time-course graphs and **H** area under the curve of extracellular taurine levels in the nAc after administration of CNO (3 mg/kg, i.p.) and vehicle (Ringer’s solution, reversed microdialysis) to rats expressing astrocyte-specific Gi-DREADDs or sham within the nAc. **G** Time-course graphs and **H** area under the curve of extracellular taurine levels in the nAc after administration of CNO and ethanol (300 mM, reversed microdialysis) to rats expressing astrocyte-specific G_i_-DREADDs or sham within the nAc. During time point 0–180 min, taurine levels in G_i_-DREADD expressing rats treated with ethanol were found to be elevated compared to vehicle-treated G_i_-DREADD rats and ethanol-treated sham rats. **I-M** Immunofluorescence images of pssAAV-2-hGFAP-HA_hM_3_D(Gq)-IRES-mCitrine-WPRE-hGHp(A) expression in the nAc. **I** Immunofluorescence staining showing neurons visualized by NeuN in blue, astrocytes expressing GFAP in green, and the DREADD conjugated with mCitrine visualized with the anti-HA tag in red (63x, scale bar 50 μm). **J-M  ***From left to right*: NeuN, GFAP, mCitrine visualized with the anti-HA tag, and a merge of all three showing the anti-HA tag to be associated with cells expressing GFAP and co-localization of GFAP and the anti-HA tag (63x, scale bar 10 μm). **N** Time-course graphs and **P** area under the curve of extracellular taurine levels in the nAc after administration of CNO (3 mg/kg, i.p.) and vehicle (Ringer’s solution, reversed microdialysis) to rats expressing astrocyte-specific G_q_-DREADDs or sham within the nAc. **O** Time-course graphs and **P** area under the curve of extracellular taurine levels in the nAc after administration of CNO and ethanol (300 mM, reversed microdialysis) to rats expressing astrocyte-specific G_q_-DREADDs or sham within the nAc. During time point 0–180 min taurine levels in G_q_-DREADD expressing rats treated with ethanol were found to be elevated compared to vehicle-treated G_i_-DREADD rats. All data are presented as mean ± SEM. *p<0.05, **p < 0.01, ***p<0.001. anti-HA = anti-hemagglutinin, AUC = area under the curve, CNO = clozapine-N-oxide, EtOH = ethanol, GFAP = glial fibrillary acidic protein, nAc = nucleus accumbens, Veh = vehicle.

Since ethanol-induced taurine elevation did not require action potential firing and was impaired during inhibition of VRACs, we hypothesized that taurine could be released from astrocytes. To specifically investigate the involvement of astrocytes in ethanol-evoked taurine release, G_i_- and G_q_-coupled DREADDs were expressed in accumbal astrocytes using the viral vector pssAAV-5/2-hGFAP-hM_4_D(Gi)_mCherry-WPRE-hGHp(A) or pssAAV-2-hGFAP-HA_hM_3_D(Gq)-IRES-mCitrine-WPRE-hGHp(A). The specificity of expression was confirmed by immunofluorescence (Fig. 6A-E). Administration of the DREADD agonist clozapine-N-oxide dihyrdrochloride (CNO; 3 mg/kg, 2 ml/kg, i.p.) to animals expressing G_i_-coupled DREADDs did not influence extracellular levels of taurine compared to rats injected with control virus (one-way ANOVA of AUC_0 − 180 min_: *F*_(3, 49)_ = 13.83, *p* < 0.001; *post hoc* analysis: G_i_(CNO + Veh) vs. Sham(CNO + Veh) *p* = 0.942; Fig. 6F, H). Furthermore, following activation of G_i_-coupled DREADDs, ethanol (300 mM) perfusion significantly increased taurine levels compared to vehicle-treated controls (*post hoc* analysis: G_i_(CNO + EtOH) vs. G_i_(CNO + Veh) *p* < 0.001; Fig. 6F-H). In fact, administration of CNO and ethanol to G_i_DREADD-expressing animals augmented the elevation of taurine compared to sham-treated rats (*post hoc* analysis: G_i_(CNO + EtOH) vs. Sham(CNO + EtOH) *p* = 0.017; Fig. 6G-H). In sham-treated controls, a significant difference was observed in the ethanol-induced taurine output (*post hoc* analysis: Sham(CNO + EtOH) vs. Sham(CNO + Veh) *p* = 0.043; Fig. 6F-H).

### Activation of astrocytes by Gq-DREADDs elevates ethanol-induced taurine increase

To selectively activate astrocytes, G_q_-coupled DREADDs were expressed in GFAP-expressing astrocytes in the nAc. The G_q_DREADD expression was confirmed by immunofluorescence (Fig. 6I-M). CNO (3 mg/kg, 2 ml/kg, i.p.) administrated to G_q_DREADD-expressing rats had no significant effect on extracellular taurine levels compared to sham-treated rats (one-way ANOVA of AUC_0 − 180 min_: *F*_(3, 44)_ = 7.40, *p* < 0.001; *post hoc* analysis: G_q_(CNO + Veh) vs. Sham(CNO + Veh) *p* = 0.459; Fig. 6N, P). When ethanol (300 mM) was perfused in the nAc, taurine levels were elevated in animals with activated G_q_DREADDs compared to vehicle-injected G_q_ controls (*post hoc* analysis: G_q_(CNO + EtOH) vs. G_q_(CNO + Veh) *p* = 0.002; Sham(CNO + EtOH) vs. Sham(CNO + Veh) *p* = 0.055; Fig. 6N-P). However, there were no enhancement in extracellular taurine output between G_q_DREADD-expressing animals compared to the sham-treated counterpart following CNO and ethanol administration (*post hoc* analysis: G_q_(CNO + EtOH) vs. Sham(CNO + EtOH) *p* = 0.914; Fig. 6O-P).

### Metabolic inhibition of astrocytes does not dampen the ethanol-induced output of taurine

**Fig. 7:**
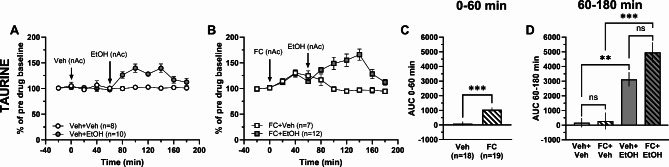
Astrocyte inhibition by fluorocitrate elevates taurine levels after ethanol. Time-course graphs of extracellular levels of **A** taurine in the nAc after ethanol (300 mM) or vehicle (Ringer’s solution) administration within the nAc using reversed microdialysis. Extracellular levels of **B** taurine in the nAc after local administration of fluorocitrate (25 µM) alone or together with ethanol. Area under the curve of **C** taurine levels during local perfusion with vehicle or fluorocitrate in the nAc during time point 0–60 min demonstrates fluorocitrate to increase extracellular taurine levels compared to Ringer-treated controls. Area under the curve of **D** taurine levels during local perfusion with vehicle, ethanol or fluorocitrate alone or together with ethanol in the nAc during time point 60–180 min shows ethanol to increase extracellular taurine levels compared to Ringer-treated controls and fluorocitrate combined with ethanol to increase extracellular taurine levels compared to fluorocitrate alone. All data are presented as mean ± SEM. **p < 0.01, ***p < 0.001. AUC = area under the curve, EtOH = ethanol, FC = fluorocitrate, nAc = nucleus accumbens, Veh = vehicle.

While our hypothesis was that ethanol-induced taurine release involved astrocytes, neither G_i_- nor G_q_-coupled DREADDs prevented the elevation, suggesting that other mechanisms are in play. However, astrocytes are highly interconnected, and may be hard to target through G-coupled receptors^42–44^. In an attempt to more completely inhibit the astrocytes, the metabolic glial inhibitor dl-fluorocitrate was used. Local perfusion with fluorocitrate (FC; 25 µM) increased taurine levels compared to vehicle (*t*-test of AUC_t=0–60 min_; *t*_35_ = 4.76, *p* < 0.001; Fig. 7A-C). Further analysis investigating local perfusion with ethanol (300 mM) in the nAc found accumbal taurine levels to be increased in vehicle treated rats (one-way ANOVA of AUC_t=60–180 min_; *F*_(3, 33)_ = 16.59, *p* < 0.001, *post hoc* analysis: Veh + EtOH vs. Veh + Veh *p* = 0.006; Fig. 7A, D). Administration of both fluorocitrate and ethanol further elevated taurine levels compared to fluorocitrate alone, with a trend towards enhanced taurine increase when compared to ethanol alone (*post hoc* analysis: FC + EtOH vs. FC + Veh *p* < 0.001, FC + EtOH vs. Veh + EtOH *p* = 0.089, Veh + Veh vs. FC + Veh *p* = 0.999; Fig. 7A-B, D).

### Nicardipine prevents ethanol-induced elevation of taurine in the nAc

**Fig. 8:**
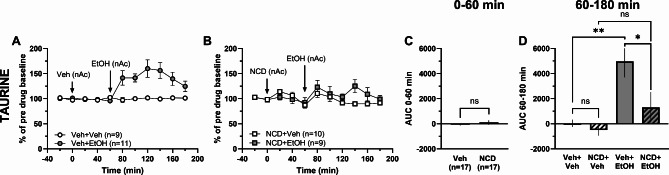
Local administration of nicardipine prevents ethanol-induced taurine output in the nAc. Time-course graphs of extracellular levels of **A** taurine in the nAc after administration of ethanol (300 mM) or vehicle (Ringer’s solution) within the nAc using reversed microdialysis. Extracellular levels of **B** taurine in the nAc following local perfusion with the L-type Ca^2+^ channel blocker nicardipine (100 µM) alone or together with ethanol. Area under the curve of **C** taurine levels during local perfusion with vehicle or nicardipine during 0–60 min demonstrates no significant difference in extracellular taurine levels between the treatment groups. Area under the curve of **D** taurine levels during local perfusion with vehicle, ethanol or nicardipine alone or together with ethanol in the nAc during time point 60–180 min shows ethanol to increase extracellular taurine levels compared to Ringer-treated controls and nicardipine combined with ethanol with no further elevation of extracellular taurine levels compared to nicardipine alone. All data are presented as mean ± SEM. *p < 0.05, **p < 0.01. AUC = area under the curve, EtOH = ethanol, NCD = nicardipine, nAc = nucleus accumbens, Veh = vehicle.

Since taurine release is suggested to be Ca^2+^ dependent^17,19^, and as ethanol has been reported to affect calcium channels^45^, the tentative Ca^2+^-dependency of ethanol-induced taurine release was assessed in the last set of experiments. Administration of the dihydropyridine L-type Ca^2+^-channel (LTCC) antagonist nicardipine (NCD; 100 µM) locally into the nAc did not affect extracellular taurine levels compared to vehicle treatment (*t*-test of AUC_t=0–60 min_: *t*_36_ = 1.00, *p* = 0.324; Fig. 8A-C). While ethanol (300 mM) in the nAc increased accumbal taurine levels in vehicle-treated controls (one-way ANOVA of AUC_t=60–180 min_: *F*_(3, 34)_ = 7.39, *p* < 0.001, *post hoc* analysis: Veh + EtOH vs. Veh + Veh *p* = 0.003; Fig. 8A, D), local ethanol administration to nicardipine-pretreated rats did not increase taurine levels compared to nicardipine treatment alone (*post hoc* analysis: NCD + EtOH vs. NCD + Veh *p* = 0.519; Fig. 8B, D). In addition, ethanol-induced taurine increase was significantly blunted in nicardipine-pretreated rats compared to vehicle (*post hoc* analysis: NCD + EtOH vs. Veh + EtOH *p* = 0.040, Veh + Veh vs. NCD + Veh *p* = 0.976; Fig. 8A-B, D).

## Discussion

The present study aimed to disentangle the mechanisms by which ethanol increases extracellular levels of taurine in the nAc, which is a pivotal step in order for ethanol to elevate dopamine levels in the same region^25^. By using in vivo microdialysis and a battery of pharmacological, chemogenetic and metabolic approaches, we aimed to establish if the ethanol-induced taurine elevation derives from accumbal astrocytes. The data presented demonstrates that the baseline levels of taurine are tonically inhibited by action potential firing and NMDA receptor activation, and that the taurine transporter plays a major role in regulating baseline extracellular taurine levels. Importantly, while inhibition of the taurine transporter or VRAC appeared to attenuate ethanol-induced taurine elevation, chemogenetic or metabolic inhibition of accumbal astrocytes rather enhanced than suppressed the extracellular rise in taurine levels following ethanol exposure. Conversely, blockade of LTCCs prevented the taurine-elevating property of ethanol, suggesting that the mechanisms involved in regulating ethanol-induced taurine output are Ca^2+^ dependent. While LTCCs may be located on both glial cells and nerve cells, our data indicate that while astrocytes are especially important for regulating baseline levels of taurine, the increased taurine levels following ethanol exposure may be originating from neurons in an action-potential-independent manner. This idea is supported by previous research, demonstrating that ablation of accumbal cholinergic interneurons inhibits ethanol-induced taurine release, without distorting basal levels of taurine^46^. A summary of all results from the different experiments are provided in Table 1.

**Table 1:** Summary of how different treatments influence taurine levels and ethanol-induced taurine levels.

	Effects on taurine output		
Treatment	Treatment vs. vehicle	Treatment + EtOH vs. Treatment	Treatment + EtOH vs. Vehicle + EtOH
**TTX** *Action potential inhibition*	↑	N/A	↑
**Memantine** *NMDAR inhibition*	↑	↑	n.s.
**GES** *Taurine transporter inhibition*	↑	n.s.	↑
**DCPIB** *VRAC inhibition*	n.s.	n.s.	n.s.
**G**_**i**_ **-DREADDs (inhibitory)** *Activation of inhibitory receptors on astrocytes*	N/A	↑	↑
**G**_**q**_ **-DREADDs (excitatory)** *Activation of excitatory receptors on astrocytes*	N/A	↑	n.s.
**FC** *Astrocyte TCA-cycle inhibition*	↑	↑	n.s.
**NCD** *LTCC inhibition*	n.s.	n.s.	**↓**

Comparison is made between treatment and vehicle (column 1), treatment + EtOH and treatment (column 2) and treatment + EtOH and vehicle + EtOH (column 3). EtOH = ethanol, FC = fluorocitrate, GES = guanidinoethyl sulfonate, DREADDs = designer receptors exclusively activated by designer drugs, LTCC = L-type Ca2+ channel, VRAC = volume regulated anion channels, TTX = tetrodotoxin, n.s.=non-significant.

Amino acids measured by in vivo microdialysis could potentially derive from both action potential-mediated exocytosis and non-exocytotic release^47^. Inhibition of action potential-mediated neurotransmission using TTX and reversed in vivo microdialysis transiently increased nAc taurine, and systemic administration of ethanol further elevated taurine levels under these circumstances. This partially unexpected finding might be connected to a reduced ability of cells to take up taurine from the extracellular space in the presence of TTX. In fact, TTX has been demonstrated to affect Na^+^/K^+^-ATPase^48^, which in turn might reduce the driving force of the Na^+^-dependent taurine transporter^1^. The increase in extracellular taurine levels following ethanol could similarly be connected to reduced clearance of taurine rather than increased release. Interestingly, other studies investigating the extracellular taurine elevation following TTX treatment demonstrate increased basal levels in the striatum^49^, a substantial reduction in the hypothalamus^50^, and TTX-independency in the ventral tegmental area (VTA)^51^. Possibly, TTX-sensitivity reflects from what source taurine is derived^49^. Since TTX inhibits voltage-gated Na^+^-channels, thereby preventing action potential-mediated exocytosis, our findings suggest that accumbal taurine most likely originates from a metabolic pool, a non-neuronal source or from neurons via a process that does not require action potential firing. This is also in agreement with previous studies in the striatum, demonstrating increased release of taurine during chelation of Ca^2+^, which is another prerequisite for action potential-mediated neurotransmitter release^52^. Here, administration of TTX did not inhibit ethanol-induced elevation of taurine levels in the nAc, as the relative taurine elevation following ethanol administration in the presence of TTX was significantly larger, confirming that this elevation does not require action potential firing^33,53^.

NMDA receptor agonists have previously been demonstrated to increase taurine levels in numerous brain regions^39^, and inhibition of the NMDA receptor using MK801 also prevents ethanol-induced taurine efflux in the hippocampus^40^. Considering the role of NMDA receptors in acute and chronic effects by ethanol exposure^54,55^, and alcohol relapse^56,57^, the NMDA receptor could possibly be a key target underlying ethanol-induced taurine release. In this study, reversed in vivo microdialysis with the NMDA receptor antagonist memantine in the nAc slightly increased extracellular levels of taurine, but did not affect the ethanol-induced taurine elevation. Thus, NMDA receptors do not appear to be a part of the mechanisms underlying ethanol-induced taurine elevation.

In this study, we also examined the role of the taurine transporter in ethanol-induced taurine increase by using the inhibitor GES. Inhibition by GES produced a substantial increase in accumbal taurine levels, and no further elevation of taurine was observed following ethanol administration in rats pre-treated with GES as compared to the corresponding control rats. While this finding suggests that ethanol affects the taurine transporter, this outcome might also be attributed to a ceiling effect. In fact, taurine does not appear to be released via the taurine transporter based on the results from Brés et al. 2000, showing that hypoosmotically-induced release of taurine is not antagonized by GES^37^. In addition, while the process by which taurine is taken up from the extracellular space by the taurine transporter is dependent on Na^+^^1^, efflux of taurine occurs independent of external Na^+^, suggesting that taurine is released via passive diffusion and not through the taurine transporter^11^. Brés et al. suggested that for basal release of taurine, there could be both an osmolarity-dependent and an osmolarity-independent component^37^. Whether this also holds true for ethanol-induced taurine release is difficult to assess. We, and others^58^, previously demonstrated that ethanol (2.5 g/kg, i.p.) diluted in a hypertonic saline solution (3.6% NaCl) prevents the ethanol-induced taurine increase in the nAc^25^, implicating an osmolarity-dependent mechanism. However, since ethanol alters the extracellular environment and induces swelling of astrocytes^34^, it is difficult to evaluate an osmolarity-independent component.

Neuronal and astrocytic cell volume is maintained via VRACs that release chloride ions and osmolytes such as taurine to regulate osmotic pressure^12–14^. Since isotonic ethanol previously has been shown to induce swelling of astrocytes and that ethanol administered in a hypertonic saline solution is unable to increase taurine^25,32–34^, we hypothesized that taurine may be released via VRACs for osmoregulation following ethanol-induced hypoosmolarity. While ethanol was unable to significantly increase taurine levels in animals pre-treated with the VRAC inhibitor DCPIB, the levels of taurine were not significantly reduced compared to vehicle-treated control rats exposed to ethanol. Consequently, the antagonism of VRACs in the nAc turned out as a trend and no complete blockade of the ethanol-mediated taurine elevation was observed. Although DCPIB is recognized as the best-in-class inhibitor of VRACs, and studies successfully show pharmacological blockade of VRACs located on astrocytes using DCPIB in the supraoptic nucleus (SON)^59^, the blocker has been reported with off-target actions toward a number of different channels and transporters and is not as specific as once assumed^60,61^. Regional differences in LRRC8-subunit composition of VRACs could be an additional or another reason why it is not possible to confirm that taurine is released from astrocytes (or neuronal VRACs) upon ethanol exposure in the nAc.

In the next set of experiments, accumbal astrocytes were targeted using a chemogenetic approach using G_i_- and G_q_-coupled DREADDs. Taurine may be considered a gliotransmitter^62^ and chemogenetic activation of astrocytes has been shown to increase Ca^2+^ in hippocampal slices and striatal cultures^63,64^, which in turn could affect gliotransmission^42,65^. While CNO activation did not affect taurine levels per se, ethanol administration induced accumbal taurine elevation in rats with either type of DREADDs following CNO activation of the receptors. Interestingly, activation of G_i_-coupled DREADD, which does not signify astrocytes being inhibited in the same way as G_i_-coupled DREADDs inhibit neurons^43,66^, in animals receiving ethanol produced an increase in taurine levels compared to the sham-injected control, whereas this difference was not found in animals expressing G_q_-coupled receptors. This finding was further partially supported by experiments where the astrocytic function was impaired using fluorocitrate, which demonstrated a trend towards increased taurine release in response to ethanol challenge. Fluorocitrate disrupts the astrocytic metabolism by uncoupling the tricarboxylic acid (TCA) cycle^67^, leading to elevated extracellular glutamate levels and decreased glutamine levels, and has been widely used in order to impair astrocytic activity^67,68^. Inhibition of the astrocytic TCA cycle resulted in an initial elevation of taurine, which might reflect impaired clearance of taurine from the extracellular space. This theory would also explain the trend towards elevated taurine levels in response to ethanol. Importantly, the sustained ethanol-induced increase in taurine suggests that astrocytes may not be the main cell type underlying ethanol-evoked elevations of taurine, but instead could be of relevance for controlling baseline levels of extracellular taurine in the nAc.

In the brain, the LTCCs are widely expressed, primarily the Ca_V_1.2 and Ca_V_1.3 subunits^69^. Both uptake and efflux of taurine has been suggested to be Ca^2+^ dependent, and dihydropyridines have been shown to be potent blockers of taurine release elicited by high levels of K^+^ or hypoosmotic solution^70–73^. Interestingly, dihydropyridines, which inhibit LTCCs, have previously been shown to reduce both the preference for ethanol^74,75^, and to inhibit the stimulatory effects by ethanol on the dopaminergic system in the rat^75^. Thus, we here investigated the influence displayed by nicardipine on ethanol-induced taurine release in the nAc. While nicardipine did not influence basal levels of extracellular taurine, it significantly inhibited ethanol-induced taurine elevation as compared to vehicle-treated controls. In fact, no difference was observed between nicardipine-pretreated rats receiving ethanol and the corresponding vehicle-control. Thus, while LTCCs do not appear to regulate basal taurine levels, they may be required for evoked release. Considering that LTCCs are distributed in all excitable and in many non-excitable cells, including vascular smooth muscle cells^76,77^, it remains to be determined where the channels required for ethanol-induced taurine elevations are located. To this end, we know that Ca_V_1.2 and Ca_V_1.3 are found on mesolimbic dopamine neurons projecting from the VTA to the nAc^78^ and that they are important for mediating dopamine signalling in the nAc^78,79^. However, the mechanistic role of these channels in relation to ethanol-induced taurine increase is unclear. Importantly, ethanol increases blood pressure^80,81^, and experimentally increased blood pressure has been found to increase the release of taurine in hypothalamus^50,82^. While we speculate that neuronal cells are important for the release of taurine, it is thus also possible that dihydropyridines act by blocking the ethanol-mediated increase in blood pressure. Future studies are required to elucidate how ethanol may influence the LTCCs leading to the increase of extracellular taurine levels and if it is LTCCs located primarily on neurons or other neuronal cells that are involved.

The data presented here show the accumbal taurine levels following ethanol exposure to be increased in a similar degree during local and systemic administration of ethanol. Importantly, systemic administration of ethanol has repeatedly been shown to reduce plasma levels of taurine, while simultaneously increasing taurine levels in the cerebrospinal fluid and interstitial fluid^27,83^. Thus, we hypothesize that accumbal taurine release is associated with local microcircuits in the nAc and does not derive from the peripheral blood stream. Ethanol-induced taurine release was partially suppressed during inhibition of VRACs showing a non-significant visual trend, indicating a role of astrocytes in ethanol-induced taurine release. However, ethanol-induced taurine increase was enhanced following chemogenetic or metabolic inhibition of astrocytes, suggesting astrocytes not being the main contributor, and also play a more prominent part in amino acid clearance. A minor role for astrocytes in ethanol-induced taurine elevation is also supported by the finding that LTCCs appear to regulate the release. In fact, the LTCC blocker verapamil has been shown to be ineffective on swelling-induced excitatory amino acid release from astrocytes or VRAC channels^84^, suggesting that taurine may be released by neurons in a manner that does not require action potential firing. While N, P and Q calcium channels are especially important for spontaneous transmitter release, the L-type channels also play a role. Interestingly, spontaneous glutamatergic neurotransmission appears less sensitive to calcium channel inhibition^85^, indicating that inhibitory neurons or cholinergic neurons may be more affected by LTCC inhibition. Indeed, the LTCC blocker nifedipine has been shown to robustly impair transmitter release from cholinergic interneurons^86^. Furthermore, accumbal ablation of cholinergic interneurons has previously been demonstrated to significantly reduce ethanol-induced taurine release, while not affecting baseline taurine levels^46^.

Notably, the study has some limitations that should be discussed. The main method used, in vivo microdialysis, is measuring a bulk-overflow of extracellular levels of taurine originating from several sources. Thus, it is not possible to identify whether the taurine is a result of neuronal release or originating from blood vessels etcetera. Another drawback is that the temporal resolution is limited and fast changes in taurine levels are not quantifiable. Consequently, future studies should use another biochemical analysis method that has a better temporal resolution. Further, when using DCPIB in order to pharmacologically target the VRACs, the vehicle itself visually elevates the taurine levels to a minor extent probably caused by the 0.4% DMSO used to dissolve DCPIB. This could potentially conceal some effects. Considering the viral transfection, in order to allow for DREADD expression, it is performed before inserting the dialysis probe. This means that there is a risk for the probe not to be inserted exactly where the DREADDs are expressed, hence some effects can be missed or lost. Further, the viral vectors used for G_i_- and G_q_-DREADDs contain two different types of fluorescent proteins, mCherry and mCitrine. Thus, when performing immunofluorescence, the expression of the DREADDs looks dissimilar and does not yield the same visual result.

In conclusion, the data presented here suggests that separate mechanisms are involved in regulating baseline levels of taurine and the ethanol-mediated taurine release. During situations where taurine clearance was impaired, such as during inhibition of astrocyte function or action potential firing, ethanol further enhanced extracellular levels, which would be expected. While astrocytes appear to play a role in regulating baseline taurine levels, we suggest that ethanol-induced taurine release primarily is associated with spontaneous release from neurons, including cholinergic interneurons. Future studies are needed to further establish the mechanisms underlying taurine release after ethanol exposure and thereby also to define its relationship to ethanol-induced dopamine release.

## Electronic supplementary material

Below is the link to the electronic supplementary material.

Supplementary Material 1

Supplementary Material 2

Supplementary Material 3

## Data Availability

The data generated during the current study are available from the corresponding author upon reasonable request.

## References

[1] Lambert, I. H., Kristensen, D. M., Holm, J. B. & Mortensen, O. H. Physiological role of taurine–From organism to organelle. *Acta Physiol. (Oxf)*. **213** (1), 191–212 (2015). 25142161 10.1111/apha.12365

[2] Decavel, C. & Hatton, G. I. Taurine immunoreactivity in the rat supraoptic nucleus: Prominent localization in glial cells. *J. Comp. Neurol. ***354** (1), 13–26 (1995). 7615871 10.1002/cne.903540103

[3] Madsen, S., Ottersen, O. P. & Storm-Mathisen, J. Immunocytochemical visualization of taurine: neuronal localization in the rat cerebellum. *Neurosci. Lett. ***60** (3), 255–260 (1985). 3906442 10.1016/0304-3940(85)90586-5

[4] Yoshida, M., Karasawa, N., Ito, M., Sakai, M. & Nagatsu, I. Demonstration of taurine-like immunoreactive structures in the rat brain. *Neurosci. Res. ***3** (4), 356–363 (1986). 2425312 10.1016/0168-0102(86)90027-1

[5] Hussy, N., Deleuze, C., Desarménien, M. G. & Moos, F. C. Osmotic regulation of neuronal activity: A new role for taurine and glial cells in a hypothalamic neuroendocrine structure. *Prog Neurobiol. ***62** (2), 113–134 (2000). 10828380 10.1016/s0301-0082(99)00071-4

[6] Huxtable, R. J. Taurine in the central nervous system and the mammalian actions of taurine. *Prog Neurobiol. ***32** (6), 471–533 (1989). 2664881 10.1016/0301-0082(89)90019-1

[7] Pasantes-Morales, H., Alavez, S., Sanchez Olea, R. & Moran, J. Contribution of organic and inorganic osmolytes to volume regulation in rat brain cells in culture. *Neurochem Res. ***18** (4), 445–452 (1993). 8097294 10.1007/BF00967248

[8] Moran, J., Maar, T. E. & Pasantes-Morales, H. Impaired cell volume regulation in taurine deficient cultured astrocytes. *Neurochem Res. ***19** (4), 415–420 (1994). 8065498 10.1007/BF00967318

[9] Vitarella, D., DiRisio, D. J., Kimelberg, H. K. & Aschner, M. Potassium and taurine release are highly correlated with regulatory volume decrease in neonatal primary rat astrocyte cultures. *J. Neurochem*. **63** (3), 1143–1149 (1994). 8051556 10.1046/j.1471-4159.1994.63031143.x

[10] Solís, J. M., Herranz, A. S., Herreras, O. & Lerma, J. Martín Del Río R. does taurine act as an osmoregulatory substance in the rat brain? *Neurosci. Lett. ***91** (1), 53–58 (1988). 3173785 10.1016/0304-3940(88)90248-0

[11] Deleuze, C., Duvoid, A. & Hussy, N. Properties and glial origin of osmotic-dependent release of taurine from the rat supraoptic nucleus. *J. Physiol. ***507** (Pt 2), 463–471 (1998). 9518705 10.1111/j.1469-7793.1998.463bt.x PMC2230788

[12] Akita, T. & Okada, Y. Characteristics and roles of the volume-sensitive outwardly rectifying (VSOR) anion channel in the central nervous system. *Neuroscience*. **275**, 211–231 (2014). 24937753 10.1016/j.neuroscience.2014.06.015

[13] Jentsch, T. J. VRACs and other ion channels and transporters in the regulation of cell volume and beyond. *Nat. Rev. Mol. Cell Biol. ***17** (5), 293–307 (2016). 27033257 10.1038/nrm.2016.29

[14] Pasantes-Morales, H., Franco, R., Torres-Marquez, M. E., Hernández-Fonseca, K. & Ortega, A. Amino acid osmolytes in regulatory volume decrease and isovolumetric regulation in brain cells: Contribution and mechanisms. *Cell. Physiol. Biochem. ***10** (5–6), 361–370 (2000). 11125217 10.1159/000016369

[15] Qiu, Z. et al. SWELL1, a plasma membrane protein, is an essential component of volume-regulated anion channel. *Cell*. **157** (2), 447–458 (2014). 24725410 10.1016/j.cell.2014.03.024 PMC4023864

[16] Voss, F. K. et al. Identification of LRRC8 heteromers as an essential component of the volume-regulated anion channel VRAC. *Science*. **344** (6184), 634–638 (2014). 24790029 10.1126/science.1252826

[17] Hyzinski-García, M. C., Rudkouskaya, A. & Mongin, A. A. LRRC8A protein is indispensable for swelling-activated and ATP-induced release of excitatory amino acids in rat astrocytes. *J. Physiol. ***592** (22), 4855–4862 (2014). 25172945 10.1113/jphysiol.2014.278887 PMC4259531

[18] Pasantes-Morales, H. & Morales Mulia, S. Influence of calcium on regulatory volume decrease: role of potassium channels. *Nephron*. **86** (4), 414–427 (2000). 11124589 10.1159/000045829

[19] McCarty, N. A. & O’Neil, R. G. Calcium signaling in cell volume regulation. *Physiol. Rev. ***72** (4), 1037–1061 (1992). 1332089 10.1152/physrev.1992.72.4.1037

[20] Di Chiara, G. & Imperato, A. Drugs abused by humans preferentially increase synaptic dopamine concentrations in the mesolimbic system of freely moving rats. *Proc. Natl. Acad. Sci. U.S.A. ***85** (14), 5274–5278 (1988). 2899326 10.1073/pnas.85.14.5274 PMC281732

[21] Wise, R. A. & Rompre, P. P. Brain dopamine and reward. *Annu. Rev. Psychol. ***40**, 191–225 (1989). 2648975 10.1146/annurev.ps.40.020189.001203

[22] Spanagel, R. Alcoholism: A systems approach from molecular physiology to addictive behavior. *Physiol. Rev. ***89** (2), 649–705 (2009). 19342616 10.1152/physrev.00013.2008

[23] Dahchour, A., Quertemont, E. & De Witte, P. Taurine increases in the nucleus accumbens microdialysate after acute ethanol administration to naive and chronically alcoholised rats. *Brain Res. ***735** (1), 9–19 (1996). 8905164 10.1016/0006-8993(96)00537-9

[24] Ericson, M. et al. Different dopamine tone in ethanol high- and low-consuming Wistar rats. *Addict. Biol. ***25** (3) e12761 (2019). 10.1111/adb.12761 31099157

[25] Ericson, M., Chau, P., Clarke, R. B., Adermark, L. & Söderpalm, B. Rising taurine and ethanol concentrations in nucleus accumbens interact to produce dopamine release after ethanol administration. *Addict. Biol. ***16** (3), 377–385 (2011). 21156034 10.1111/j.1369-1600.2010.00245.x

[26] Olive, M. F. Interactions between taurine and ethanol in the central nervous system. *Amino Acids*. **23** (4), 345–357 (2002). 12436202 10.1007/s00726-002-0203-1

[27] Ulenius, L., Andrén, A., Adermark, L., Söderpalm, B. & Ericson, M. Sub-chronic taurine administration induces behavioral sensitization but does not influence ethanol-induced dopamine release in the nucleus accumbens. *Pharmacol. Biochem. Behav. ***188**, 172831 (2020). 31770542 10.1016/j.pbb.2019.172831

[28] Ademar, K., Adermark, L., Söderpalm, B. & Ericson, M. Sodium acamprosate and calcium exert additive effects on nucleus accumbens dopamine in the rat. *Addict. Biol. ***27** (5), e13224 (2022). 36001425 10.1111/adb.13224 PMC9541434

[29] Chau, P., Lido, H. H., Soderpalm, B. & Ericson, M. Acamprosate’s ethanol intake-reducing effect is associated with its ability to increase dopamine. *Pharmacol. Biochem. Behav. ***175**, 101–107 (2018). 30266455 10.1016/j.pbb.2018.09.009

[30] Boismare, F. et al. A homotaurine derivative reduces the voluntary intake of ethanol by rats: are cerebral GABA receptors involved? *Pharmacol. Biochem. Behav. ***21** (5), 787–789 (1984). 6096898 10.1016/s0091-3057(84)80020-9

[31] Olive, M. F., Nannini, M. A., Ou, C. J., Koenig, H. N. & Hodge, C. W. Effects of acute acamprosate and homotaurine on ethanol intake and ethanol-stimulated mesolimbic dopamine release. *Eur. J. Pharmacol. ***437** (1–2), 55–61 (2002). 11864639 10.1016/s0014-2999(02)01272-4

[32] Adermark, L. et al. Implications for glycine receptors and astrocytes in ethanol-induced elevation of dopamine levels in the nucleus accumbens. *Addict. Biol. ***16** (1), 43–54 (2011). 20331561 10.1111/j.1369-1600.2010.00206.x

[33] Kimelberg, H. K. et al. Ethanol-induced aspartate and taurine release from primary astrocyte cultures. *J. Neurochem*. **60** (5), 1682–1689 (1993). 8473890 10.1111/j.1471-4159.1993.tb13391.x

[34] Allansson, L., Khatibi, S., Olsson, T. & Hansson, E. Acute ethanol exposure induces [Ca2+]i transients, cell swelling and transformation of actin cytoskeleton in astroglial primary cultures. *J. Neurochem*. **76** (2), 472–479 (2001). 11208910 10.1046/j.1471-4159.2001.00097.x

[35] Pasantes-Morales, H., Moran, J. & Schousboe, A. Volume-sensitive release of taurine from cultured astrocytes: properties and mechanism. *Glia*. **3** (5), 427–432 (1990). 2146228 10.1002/glia.440030514

[36] Cardin, V., Pena-Segura, C. & Pasantes-Morales, H. Activation and inactivation of taurine efflux in hyposmotic and isosmotic swelling in cortical astrocytes: Role of ionic strength and cell volume decrease. *J. Neurosci. Res. ***56** (6), 659–667 (1999). 10374821 10.1002/(SICI)1097-4547(19990615)56:6<659::AID-JNR12>3.0.CO;2-W

[37] Brès, V. et al. Pharmacological characterization of volume-sensitive, taurine permeable anion channels in rat supraoptic glial cells. *Br. J. Pharmacol. ***130** (8), 1976–1982 (2000). 10952690 10.1038/sj.bjp.0703492 PMC1572259

[38] Paxinos, G. & Watson, C. *The Rat Brain in Stereotaxic Coordinates*. Figure 18. 6 edn (eds Watson, C.) (Elsevier, Academic, 2007).

[39] Saransaari, P. & Oja, S. S. Excitatory amino acids evoke taurine release from cerebral cortex slices from adult and developing mice. *Neuroscience*. **45** (2), 451–459 (1991). 1684837 10.1016/0306-4522(91)90240-o

[40] Lallemand, F., Dahchour, A., Ward, R. J. & De Witte, P. Does taurine play an osmolarity role during ethanol intoxication? *Adv. Exp. Med. Biol. ***483**, 203–212 (2000). 11787599 10.1007/0-306-46838-7_22

[41] Hölter, S. M., Danysz, W. & Spanagel, R. Evidence for alcohol anti-craving properties of memantine. *Eur. J. Pharmacol. ***314** (3), R1–R2 (1996). 8957265 10.1016/s0014-2999(96)00670-x

[42] Shen, W. et al. Chemogenetic manipulation of astrocytic activity: is it possible to reveal the roles of astrocytes? *Biochem. Pharmacol. ***186**, 114457 (2021). 33556341 10.1016/j.bcp.2021.114457

[43] Lee, S. H., Mak, A. & Verheijen, M. H. G. Comparative assessment of the effects of DREADDs and endogenously expressed GPCRs in hippocampal astrocytes on synaptic activity and memory. *Front. Cell. Neurosci. ***17**, 1159756 (2023). 37051110 10.3389/fncel.2023.1159756 PMC10083367

[44] Adermark, L. & Lovinger, D. M. Ethanol effects on electrophysiological properties of astrocytes in striatal brain slices. *Neuropharmacology*. **51** (7–8), 1099–1108 (2006). 16938316 10.1016/j.neuropharm.2006.05.035

[45] Walter, H. J., McMahon, T., Dadgar, J., Wang, D. & Messing, R. O. Ethanol regulates calcium channel subunits by protein kinase C delta -dependent and -independent mechanisms. *J. Biol. Chem. ***275** (33), 25717–25722 (2000). 10835432 10.1074/jbc.M910282199

[46] Loftén, A., Adermark, L., Ericson, M. & Söderpalm, B. Regulation of ethanol-mediated dopamine elevation by glycine receptors located on cholinergic interneurons in the nucleus accumbens. *Addict. Biol. ***28** (12), e13349 (2023). 38017639 10.1111/adb.13349

[47] Timmerman, W. & Westerink, B. H. Brain microdialysis of GABA and glutamate: what does it signify? *Synapse*. **27** (3), 242–261 (1997). 9329159 10.1002/(SICI)1098-2396(199711)27:3<242::AID-SYN9>3.0.CO;2-D

[48] Reznik, L. V. & Miazina, E. M. [Inhibition of Na+, K+-ATPase activity by sodium channel blockers]. *Vopr Med. Khim. ***31** (4), 122–124 (1985). 2413621

[49] Molchanova, S., Oja, S. S. & Saransaari, P. Characteristics of basal taurine release in the rat striatum measured by microdialysis. *Amino Acids*. **27** (3–4), 261–268 (2004). 15549491 10.1007/s00726-004-0139-8

[50] Singewald, N., Guo, L. J. & Philippu, A. Taurine release in the hypothalamus is altered by blood pressure changes and neuroactive drugs. *Eur. J. Pharmacol. ***240** (1), 21–27 (1993). 8104813 10.1016/0014-2999(93)90540-x

[51] Timmerman, W., Cisci, G., Nap, A., de Vries, J. B. & Westerink, B. H. Effects of handling on extracellular levels of glutamate and other amino acids in various areas of the brain measured by microdialysis. *Brain Res. ***833** (2), 150–160 (1999). 10375690 10.1016/s0006-8993(99)01538-3

[52] Della Corte, L., Bolam, J. P., Clarke, D. J., Parry, D. M. & Smith, A. D. Sites of [3H]taurine uptake in the rat substantia Nigra in relation to the release of taurine from the striatonigral pathway. *Eur. J. Neurosci. ***2** (1), 50–61 (1990). 12106102 10.1111/j.1460-9568.1990.tb00380.x

[53] Aschner, M., Vitarella, D., Allen, J. W., Conklin, D. R. & Cowan, K. S. Methylmercury-induced inhibition of regulatory volume decrease in astrocytes: Characterization of osmoregulator efflux and its reversal by amiloride. *Brain Res. ***811** (1–2), 133–142 (1998). 9804925 10.1016/s0006-8993(98)00629-5

[54] Lovinger, D. M., White, G. & Weight, F. F. Ethanol inhibits NMDA-activated ion current in hippocampal neurons. *Science*. **243** (4899), 1721–1724 (1989). 2467382 10.1126/science.2467382

[55] Hoffman, P. L. et al. Ethanol and the NMDA receptor. *Alcohol*. **7** (3), 229–231 (1990). 2158789 10.1016/0741-8329(90)90010-a

[56] Tsai, G. & Coyle, J. T. The role of glutamatergic neurotransmission in the pathophysiology of alcoholism. *Annu. Rev. Med. ***49**, 173–184 (1998). 9509257 10.1146/annurev.med.49.1.173

[57] Gass, J. T. & Olive, M. F. Glutamatergic substrates of drug addiction and alcoholism. *Biochem. Pharmacol. ***75** (1), 218–265 (2008). 17706608 10.1016/j.bcp.2007.06.039 PMC2239014

[58] Quertemont, E., Devitgh, A. & De Witte, P. Systemic osmotic manipulations modulate ethanol-induced taurine release: A brain microdialysis study. *Alcohol*. **29** (1), 11–19 (2003). 12657372 10.1016/s0741-8329(02)00324-5

[59] Choe, K. Y., Olson, J. E. & Bourque, C. W. Taurine release by astrocytes modulates osmosensitive glycine receptor tone and excitability in the adult supraoptic nucleus. *J. Neurosci. ***32** (36), 12518–12527 (2012). 22956842 10.1523/JNEUROSCI.1380-12.2012 PMC6621246

[60] Afzal, A. et al. The LRRC8 volume-regulated anion channel inhibitor, DCPIB, inhibits mitochondrial respiration independently of the channel. *Physiol. Rep. ***7** (23), e14303 (2019). 31814333 10.14814/phy2.14303 PMC6900491

[61] Bowens, N. H., Dohare, P., Kuo, Y-H. & Mongin, A. A. DCPIB, the proposed selective blocker of volume-regulated anion channels, inhibits several glutamate transport pathways in glial cells. *Mol. Pharmacol. ***83** (1), 22–32 (2013). 23012257 10.1124/mol.112.080457 PMC3533478

[62] Ramírez-Guerrero, S. et al. Taurine and astrocytes: A homeostatic and neuroprotective relationship. *Front. Mol. Neurosci. ***15**, 937789 (2022). 35866158 10.3389/fnmol.2022.937789 PMC9294388

[63] Agulhon, C. et al. Modulation of the autonomic nervous system and behaviour by acute glial cell gq protein-coupled receptor activation in vivo. *J. Physiol. ***591** (22), 5599–5609 (2013). 24042499 10.1113/jphysiol.2013.261289 PMC3853498

[64] Bull, C. et al. Rat nucleus accumbens core astrocytes modulate reward and the motivation to self-administer ethanol after abstinence. *Neuropsychopharmacology*. **39** (12), 2835–2845 (2014). 24903651 10.1038/npp.2014.135 PMC4200494

[65] Zorec, R. et al. Astroglial excitability and gliotransmission: an appraisal of Ca2 + as a signalling route. *ASN Neuro ***4**(2). (2012). 10.1042/AN20110061 PMC3310306 22313347

[66] Durkee, C. A. et al. Gi/o protein-coupled receptors inhibit neurons but activate astrocytes and stimulate gliotransmission. *Glia*. **67** (6), 1076–1093 (2019). 30801845 10.1002/glia.23589 PMC6462242

[67] Fonnum, F., Johnsen, A. & Hassel, B. Use of fluorocitrate and fluoroacetate in the study of brain metabolism. *Glia*. **21** (1), 106–113 (1997). 9298853

[68] Adermark, L. et al. Astrocytes modulate extracellular neurotransmitter levels and excitatory neurotransmission in dorsolateral striatum via dopamine D2 receptor signaling. *Neuropsychopharmacology*. **47** (8), 1493–1502 (2022). 34811469 10.1038/s41386-021-01232-x PMC9206030

[69] Striessnig, J., Pinggera, A., Kaur, G., Bock, G. & Tuluc, P. L-type ca(2+) channels in heart and brain. *Wiley Interdiscip Rev. Membr. Transp. Signal. ***3** (2), 15–38 (2014). 24683526 10.1002/wmts.102 PMC3968275

[70] Sánchez-Olea, R., Morales Mulia, M., Morán, J. & Pasantes-Morales, H. Inhibition by dihydropyridines of regulatory volume decrease and osmolyte fluxes in cultured astrocytes is unrelated to extracellular calcium. *Neurosci. Lett. ***193** (3), 165–168 (1995). 7478174 10.1016/0304-3940(95)11691-o

[71] Li, G., Liu, Y. & Olson, J. E. Calcium/calmodulin-modulated chloride and taurine conductances in cultured rat astrocytes. *Brain Res. ***925** (1), 1–8 (2002). 11755895 10.1016/s0006-8993(01)03235-8

[72] Philibert, R. A. & Dutton, G. R. Dihydropyridines modulate K+-evoked amino acid and adenosine release from cerebellar neuronal cultures. *Neurosci. Lett. ***102** (1), 97–102 (1989). 2476690 10.1016/0304-3940(89)90314-5

[73] Philibert, R. A., Rogers, K. L. & Dutton, G. R. Stimulus-coupled taurine efflux from cerebellar neuronal cultures: on the roles of ca + + and Na+. *J. Neurosci. Res. ***22** (2), 167–171 (1989). 2468785 10.1002/jnr.490220209

[74] De Beun, R., Schneider, R., Klein, A., Lohmann, A. & De Vry, J. Effects of nimodipine and other calcium channel antagonists in alcohol-preferring AA rats. *Alcohol*. **13** (3), 263–271 (1996). 8734841 10.1016/0741-8329(95)02054-3

[75] Engel, J. A. et al. Biochemical and behavioral evidence for an interaction between ethanol and calcium channel antagonists. *J. Neural Transm*. **74** (3), 181–193 (1988). 3210013 10.1007/BF01244784

[76] Tsien, R. W., Ellinor, P. T. & Horne, W. A. Molecular diversity of voltage-dependent Ca2 + channels. *Trends Pharmacol. Sci. ***12** (9), 349–354 (1991). 1659003 10.1016/0165-6147(91)90595-j

[77] D’Ascenzo, M. et al. Electrophysiological and molecular evidence of L-(Cav1), N- (Cav2.2), and R- (Cav2.3) type Ca2+ channels in rat cortical astrocytes. *Glia*. **45** (4), 354–363 (2004). 14966867 10.1002/glia.10336

[78] Liu, Y. et al. Cav1.2 and Cav1.3 L-type calcium channels regulate dopaminergic firing activity in the mouse ventral tegmental area. *J. Neurophysiol. ***112** (5), 1119–1130 (2014). 24848473 10.1152/jn.00757.2013 PMC4122730

[79] Nunes, E. J. & Addy, N. A. L-type calcium channel regulation of dopamine activity in the ventral tegmental area to nucleus accumbens pathway: Implications for substance use, mood disorders and co-morbidities. *Neuropharmacology*. **224**, 109336 (2023). 36414149 10.1016/j.neuropharm.2022.109336 PMC11215796

[80] Kawano, Y. Physio-pathological effects of alcohol on the cardiovascular system: its role in hypertension and cardiovascular disease. *Hypertens. Res. ***33** (3), 181–191 (2010). 20075936 10.1038/hr.2009.226

[81] Riff, D. P., Jain, A. C. & Doyle, J. T. Acute hemodynamic effects of ethanol on normal human volunteers. *Am. Heart J. ***78** (5), 592–597 (1969). 5348743 10.1016/0002-8703(69)90510-9

[82] Philippu, A. Brain mapping: Topography of neurons and their transmitters involved in various brain functions. *Naunyn. Schmiedebergs Arch. Pharmacol. ***396** (7), 1415–1422 (2023). 37184687 10.1007/s00210-023-02523-4

[83] Abdel-Nabi, R., Milakofsky, L., Hofford, J. M., Hare, T. A. & Vogel, W. H. Effect of ethanol on amino acids and related compounds in rat plasma, heart, aorta, bronchus, and pancreas. *Alcohol*. **13** (2), 171–174 (1996). 8814652 10.1016/0741-8329(95)02031-4

[84] Abdullaev, I. F., Rudkouskaya, A., Schools, G. P., Kimelberg, H. K. & Mongin, A. A. Pharmacological comparison of swelling-activated excitatory amino acid release and Cl- currents in cultured rat astrocytes. *J. Physiol. ***572** (Pt 3), 677–689 (2006). 16527858 10.1113/jphysiol.2005.103820 PMC1780004

[85] Williams, C. L. & Smith, S. M. Calcium dependence of spontaneous neurotransmitter release. *J. Neurosci. Res. ***96** (3), 335–347 (2018). 28699241 10.1002/jnr.24116 PMC5766384

[86] Losavio, A. & Muchnik, S. Role of L-type and N-type voltage-dependent calcium channels (VDCCs) on spontaneous acetylcholine release at the mammalian neuromuscular junction. *Ann. N. Y. Acad. Sci. ***841**, 636–645 (1998). 9668307 10.1111/j.1749-6632.1998.tb10995.x

