# Supplementary figures and images for: Separate mechanisms regulating accumbal taurine levels during baseline conditions and following ethanol exposure in the rat
Source: Sci Rep. 2024 Oct 15;14:24166. doi: 10.1038/s41598-024-74449-7 (PMC11480114; doi:10.1038/s41598-024-74449-7)

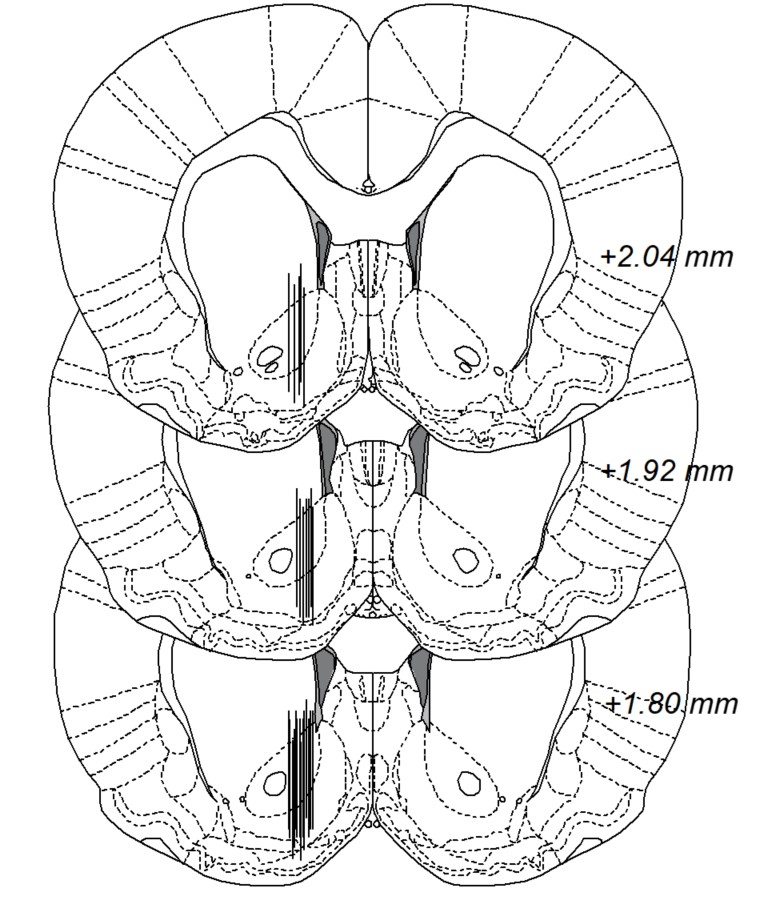

Supplement: Supplementary file 1 — Supplementary Material 1 [file 41598_2024_74449_MOESM1_ESM.tif]
