# Supplementary material for: Separate mechanisms regulating accumbal taurine levels during baseline conditions and following ethanol exposure in the rat
Source: Sci Rep. 2024 Oct 15;14:24166. doi: 10.1038/s41598-024-74449-7 (PMC11480114; doi:10.1038/s41598-024-74449-7)
Supplement: Supplementary file 2 — Supplementary Material 2 [file 41598_2024_74449_MOESM2_ESM.docx]

# LEGENDS

**Fig. S1 Histology.** Representation of microdialysis probe placement in the nAc. The black lines illustrate traces from a selection of rats included in the study. Adjacent numbers indicate distance from bregma.
