# Supplementary material for: Separate mechanisms regulating accumbal taurine levels during baseline conditions and following ethanol exposure in the rat
Source: Sci Rep. 2024 Oct 15;14:24166. doi: 10.1038/s41598-024-74449-7 (PMC11480114; doi:10.1038/s41598-024-74449-7)
Supplement: Supplementary file 3 — Supplementary Material 3 [file 41598_2024_74449_MOESM3_ESM.docx]

# Separate mechanisms regulating accumbal taurine levels during baseline conditions and following ethanol exposure in the rat

### Karin Ademar ^1*^, Lisa Ulenius^1^, Anna Loftén^1,3^, Bo Söderpalm^1,3^, Louise Adermark^1,2^, and Mia Ericson^1^

^1^ Addiction Biology Unit, Department of Psychiatry and Neurochemistry, Institute of Neuroscience and Physiology, Sahlgrenska Academy, University of Gothenburg, Gothenburg, Sweden

^2^ Department of Pharmacology, Institute of Neuroscience and Physiology, Sahlgrenska Academy, University of Gothenburg, Gothenburg, Sweden

^3^ Beroendekliniken, Sahlgrenska University Hospital, Gothenburg, Sweden

***Corresponding author:**

Karin Ademar

Addiction Biology Unit

Department of Psychiatry and Neurochemistry

Institute of Neuroscience and Physiology

Sahlgrenska Academy

Box 410

SE 405 30 Gothenburg

Sweden

E-mail: karin.ademar@gu.se

Telephone: +46 31 786 39 77

# **SUPPLEMENTARY INFORMATION**

## **METHODS AND MATERIALS**

### Animals

Male Wistar rats (Taconic, Denmark or Envigo/Inotiv, The Netherlands; n=347) were group housed from arrival until surgery for microdialysis probe placement. The rats were kept at a 12-hour light/dark cycle (lights on at 7:00 AM; lights off at 7:00 PM) with food and water *ad libitum*. All rats were allowed one week of acclimatization to the animal facility (room temperature 20-22°C, relative humidity 50-65%) before any experiments were initiated. The experiments were approved by the Ethics Committee for Animal Experiments in Gothenburg, Sweden (2401/19; 3095/20), followed the Swedish National Committee for Animal Research guidelines and is reported in accordance with ARRIVE guidelines.

### Drugs

Ethanol (EtOH; 95%; Kemetyl AB, Haninge, Sweden or Kiilto Clean AB, Täby, Sweden) was diluted in saline (0.9% NaCl) to a concentration of 15% and administrated intraperitoneally (i.p.; 2.5 g/kg, 15 ml/kg) or diluted in Ringer’s solution (consisting of (in mmol/l): 140 NaCl, 1.2 CaCl_2_, 3.0 KCl and 1.0 MgCl_2_) to a concentration of 300 mM and perfused via the microdialysis probe. The action potential inhibitor and sodium channel blocker tetrodotoxin (TTX; Tocris, Bristol, UK) was dissolved in Ringer’s solution and diluted to a concentration of 1 μM (local perfusion via the microdialysis probe). The non-competitive NMDA receptor antagonist memantine (Mem; Sigma-Aldrich, Stockholm, Sweden) was dissolved in Ringer’s solution and diluted to a concentration of 100 μM (local perfusion via the microdialysis probe). The competitive taurine transporter inhibitor guanidinoethyl sulfonate (GES; Toronto Research Chemicals, Toronto, Canada) was dissolved in Ringer’s solution and diluted to a concentration of 5 mM (local perfusion via the microdialysis probe). The volume-regulated anion channel (VRAC) blocker DCPIB (oxobutyric acid; Tocris, Bristol, UK) was dissolved in dimethyl sulfoxide (DMSO) and diluted in Ringer’s solution to a concentration of 100 μM (final concentration of DMSO 0.4% for both DCPIB- and the corresponding vehicle rats; local perfusion via the microdialysis probe). The concentration of DCPIB was attempted to be increased by using a higher percentage of DMSO, however this was not successful as DMSO alone had a major impact on taurine. The specific DREADD ligand clozapine-N-oxide dihyrdrochloride (CNO; Tocris, Bristol, UK) was dissolved in saline and administrated i.p. (3 mg/kg, 2 ml/kg). The metabolic uncoupler dl-fluorocitrate (FC; Sigma-Aldrich, Stockholm, Sweden) was dissolved in Ringer´s solution and diluted to a concentration of 25 μM (local perfusion via the microdialysis probe). Finally, the L-type Ca^2+^ channel (LTCC) antagonist nicardipine (NCD; Sigma-Aldrich, Stockholm, Sweden) was dissolved in pre-warmed Ringer’s solution and diluted to a concentration of 100 μM (local perfusion via the microdialysis probe).

### Viral microinjection of DREADDs targeting astrocytes

To more selectively target astrocytes we expressed G_i_- and G_q_-coupled designer receptors exclusively activated by designer drugs (DREADDs) targeting the astrocyte specific protein glial fibrillary acidic protein (GFAP) locally in nAc. Rats (n=101, weighing 190-210 g) were anesthetized by isoflurane (Baxter, Kista, Sweden), mounted onto a stereotaxic instrument (David Kopf Instruments, AgnTho’s, Lidingö, Sweden), and placed on a heating pad to prevent hypothermia. The skull was exposed and a hole was drilled for unilateral injection of the viral vector (pssAAV-2-hGFAP-HA_hM_3_D(Gq)-IRES-mCitrine-WPRE-hGHp(A) (GFAP-Gq-DREADD), (physical titer: 4.6x10^12^ vg/ml); pssAAV-5/2-hGFAP-hM_4_D(Gi)_mCherry-WPRE-hGHp(A) (GFAP-Gi-DREADD), (physical titer: 4.7x10^12^ vg/ml) or pssAAV-2-hGFAP-EGFP-WPRE-hGHp(A) (sham), (physical titer: 1.8x10^13^ vg/ml); Viral Vector Facility (VVF), Neuroscience Center Zurich (ZNZ), Zurich, Switzerland). Using a 10 µl Hamilton syringe attached to a 31-gauge microinjection cannula (AMI-5T, AgnTho’s AB, Lidingö, Sweden), 0.8 μl of the viral vector was infused into the nAc core/shell borderline region (AP: +1.5 mm, ML: -1.4 mm relative to bregma and DV: -7.8 mm relative to the scull (1)) at 0.05 μl per minute with the aid of a microinfusion pump (U-864 Syringe Pump, AgnTho’s, Lidingö, Sweden). The cannula was withdrawn 5 minutes after completed infusion to allow for diffusion. The skin was sealed with surgical clips and the rats received postoperative analgesia (Norocarp, 5 mg/kg, s.c) before returning to their home cage for three weeks prior to implantation of an *in vivo* microdialysis probe or immunofluorescence staining.

### *In vivo* microdialysis and biochemical assays

Two days prior to the *in vivo* microdialysis experiment, rats (weighing 280-360 g corresponding to an age of 9-10 weeks) were anesthetized by isoflurane, mounted onto a stereotaxic instrument, and placed on a heating pad to prevent hypothermia. To provide analgesia during and after surgery and prevent dehydration, rats received Metacam® (1 mg/ml, 2 ml/kg; Boehringer Ingelheim, Ingelheim/Rhein, Germany) subcutaneously (s.c.), Marcain® (Aspen Pharma, Dublin, Ireland) infiltrated alongside the surgical incision and a s.c. injection with saline (2 ml; 0,9% NaCl). For viral injected rats, the surgical clips were removed, and two holes were drilled for anchoring screws. For treatment-naïve rats, a third hole was drilled for probe placement. A dialysis probe, custom-made in the laboratory, with a 2 mm active space and a 20 kDa molecular cut-off was lowered into the nAc core/shell borderline region (AP: +1.85 mm, ML: -1.4 mm relative to bregma and DV: -7.8 mm relative to dura mater (1)). The dialysis probe was fixed to the skull using two anchoring screws and dental cement (DAB Dental AB, Gothenburg, Sweden). Animals were single-housed during the 48-hour surgical recovery.

On the day of the *in vivo* microdialysis experiment, the inlet and outlet of the microdialysis probe were connected to a microperfusion pump via a swivel, allowing the animal to move around freely in its home cage. The probe was perfused with Ringer’s solution at a rate of 2 μl per minute for two hours prior to baseline sampling to allow for equilibration. Dialysate samples (40 μl) were collected every 20 minutes. Drug administration, via i.p. injection or local perfusion by reversed dialysis, was initiated when four stable baseline samples (±10%) were obtained. Immediately after termination of the experiment, the rats were decapitated and the brains dissected, fixed (Accustain, Sigma-Aldrich, Stockholm, Sweden) and stored (4°C) for 4-7 days until further probe placement verification. Rats with incorrect probe placement, visual signs of bleeding or other visual defects were excluded from the statistical analysis (n=23; see Fig. S1 for probe placements).

### Biochemical assays

For separation and detection of taurine, a high performance liquid chromatography (HPLC) system with fluorescence detection was used as previously described (2). External standards containing 0.5 and 1.0 μM taurine were used to identify and quantify the concentration. Before analysis, in order to maintain stability of the samples, sodium azide (50% v/v) was added to each sample. The chromatogram was analyzed using Thermo Scientific Chromeleon Chromatography Data System software (CHROMELEON7).

### Immunofluorescence

Rats were deeply anesthetized with Allfatal® (350 mg/kg, 100 mg/ml, Omnidea, Apoteket AB, Sweden) i.p. and were transcardially perfused with 100 ml buffer (116 mM NaCl, 5.4 mM KCl, 1.6 mM MgCl_2_, 0.4 mM MgSO_4_, 1.3 mM NaH_2_PO_4_, 26 mM NaHCO_3_, 5.5 mM glucose) and 4% paraformaldehyde (300 ml). The brains were post fixed (4% paraformaldehyde, 90 minutes, 4°C), cryoprotected (10% sucrose, 24 h, 4°C followed by 20%, 1 week, 4°C) and snap-frozen using isopentane (Sigma-Aldrich, Stockholm, Sweden) on dry ice and stored at -80°C. Coronal slices (40 μm) were sectioned using a Leica CM1950 cryostat (Leica Biosystems, Nussloch, Germany) and placed in cryoprotective medium (30% glycerol, 30% ethylene glycol, 40% 1xTris-buffered saline [TBS; 0.15 M NaCl, 0.05 M Tris-HCl; pH 7.6]) at -20°C. Brain sections were washed in 1xTBS for 3x10 min and blocked for 1 h in blocking solution (1% Triton X-100, 3% normal donkey serum (Jackson ImmunoResearch, West Grove, PA, USA), in 1xTBS). Next, the sections were incubated with primary antibody in blocking solution (1:1000 anti-hemagglutinin (anti-HA; Nordic Biosite, Täby, Sweden), 1:1000 anti-GFAP (Invitrogen, Thermo Scientific, Gothenburg, Sweden) and 1:400 anti-NeuN (Millipore Merck, Solna, Sweden) in combination or 1:1000 anti-GFAP and 1:400 anti-NeuN in combination) overnight at 4°C and in dark conditions. Following primary incubation, the sections were washed in 1xTBS for 3x10 min and incubated with secondary antibody in blocking solution (1:1000 Alexa Flour 488 Donkey Anti-mouse, Alexa Flour 555 Donkey Anti-sheep and Alexa Flour 647 Donkey Anti-rabbit in combination or 1:1000 Alexa Fluor 488 Donkey Anti-mouse and Alexa Fluor 647 Donkey Anti-rabbit in combination (Invitrogen, Thermo Scientific, Gothenburg, Sweden)) for one hour at room temperature. The sections were washed in 1xTBS for 3x10 min, mounted onto microscope slides, coverslipped with Fluoroshield (Sigma-Aldrich, Stockholm, Sweden) and dried overnight in room temperature. Images were obtained with a Zeiss LSM 700 inverted confocal microscope (Zeiss, Jena, Germany).

### Statistics

Data were analyzed using GraphPad Prism, version 10 for Windows (GraphPad Software, Inc., San Diego, CA, USA). Two-way analysis of variance (ANOVA) with repeated measures (treatment group x time) over the relevant period of time followed by Tukey’s multiple comparisons test was used for statistical analysis of animals receiving ethanol treatment only. For all the other microdialysis experiments, area under the curve (AUC) over the relevant period of time was calculated for each individual animal and one-way ANOVA followed by Tukey’s post hoc analysis or unpaired *t*-test was used to analyze significant differences in AUC between treatment groups. The relevant period of time was based on when the drug used for pre-treatment yielded stable taurine levels, which then was decisive for when ethanol was to be administered. All data are presented as mean ± standard error of the mean (SEM) and a probability value (*p*) less than 0.05 was considered to be statistically significant.

## **REFERENCES**

1. Paxinos G, Watson C. The rat brain in stereotaxic coordinates. 6. ed. ed. Watson C, editor. Amsterdam [u.a.]: Amsterdam u.a. : Elsevier, Academic Press; 2007.

2. Ulenius L, Andrén A, Adermark L, Söderpalm B, Ericson M. Sub-chronic taurine administration induces behavioral sensitization but does not influence ethanol-induced dopamine release in the nucleus accumbens. Pharmacol Biochem Behav. 2020;188:172831.
